# Effect of HIgh-Flow Therapy in Long-Term Oxygen Therapy (HILOT): study protocol for a multicentre, registry-based, randomised clinical trial

**DOI:** 10.1186/s13063-026-09488-8

**Published:** 2026-02-04

**Authors:** Josefin Sundh, Mirjam Ljunggren, Andreas Palm, Eva Lindberg, Florent Lavergne, Ulla Møller Weinreich, Zainab Ahmadi, Magnus Ekström, the HILOT Collaboration Group, Anders Blomberg, Pia Ghosh, Taivo Kipper, Ozran Kricka, Bo Pedersen, Anders Andersson, Jonas Einarsson, Hanan Tanash

**Affiliations:** 1 Department of Respiratory Medicine, Faculty of Medicine and Health, Örebro University, Örebro, Sweden; 2 Department of Medical Science, Respiratory-, Allergy- and Sleep Research, Uppsala University, Uppsala, Sweden; 3 Université Pierre et Marie Curie, Paris, France; 4 Department of Respiratory Diseases, Aalborg University Hospital, Respiratory Research Aalborg, Aalborg University, Aalborg, Denmark; 5 Department of Respiratory Medicine, Allergology and Palliative Medicine, Institution for Clinical Sciences in Lund, Lund University, Lund, Sweden

**Keywords:** Long-term oxygen therapy, Chronic obstructive pulmonary disease, Interstitial lung disease, Low-flow oxygen therapy, High-flow oxygen therapy, Exacerbations, Hospitalisations, Mortality, Symptoms, Adverse events

## Abstract

**Background:**

The use of high-flow oxygen therapy (HFOT) compared with standard low-flow oxygen therapy (LFOT) may improve outcomes in people with oxygen-dependent chronic respiratory failure (CRF). The primary aim of this multicentre trial was to evaluate HFOT in addition to LFOT, compared with regular LFOT in people with CRF due to chronic obstructive pulmonary disease (COPD) or interstitial lung disease (ILD).

**Methods:**

Registry-based randomised controlled trial (R-RCT) of people on LFOT for CRF due to COPD (*n* = 270) and ILD (*n* = 40), at ten Swedish secondary care centres within the Swedish Register for Respiratory Failure (Swedevox). People with ongoing LFOT are randomised in a 1:1 ratio to standard treatment with LFOT (control) or LFOT with added HFOT during nighttime and at the patient’s discretion daytime (intervention). HFOT is provided using the ResMed Lumis HFT system and the AcuCare HFNC Cannula. Primary outcome is time to first hospitalisation or death up to 1 year in people with COPD. Secondary outcomes include symptoms, health-related outcomes (HRQL), health-economics, adverse events, and to explore the effects of HFOT in people with CRF due to ILD. Outcome data will be obtained from national registries and from patient questionnaires at 3 and 12 months.

**Discussion:**

This R-RCT will combine the advantages of a prospective randomised trial and large clinical national registries to improve the evidence-based use of long-term oxygen therapy. Recruitment started in June 2024 and is ongoing.

**Trial registration:**

ClinicalTrials.gov, ID: NCT06247397. Registered 2024–02–07.

**Supplementary Information:**

The online version contains supplementary material available at 10.1186/s13063-026-09488-8.

## Administrative information

**Table:** 

Title {1}	Effect of HIgh-flow Therapy in Long-term Oxygen Therapy (HILOT): Study protocol of a multicentre, registry-based, randomised clinical trial
Trial registration {2a and 2b}	Registered on clinicaltrials.gov 2024–02–07: NCT06247397
Protocol version {3}	Version No: 1.1, date: 2025–02–21
Funding {4}	Grants from Swedish Research Council (2019–02081, Magnus Ekström), Swedish Respiratory Society (SLS-985470, Josefin Sundh) and ResMed High-flow oxygen therapy (HFOT) equipment and training is provided by ResMed
Author details {5a}	JS: Department of Respiratory Medicine, Faculty of Medicine and Health, Örebro University, Örebro, Sweden ML, AP, EL: Department of Medical science, Respiratory-, allergy- and sleep research, Uppsala University, Uppsala, Sweden FL: Université Pierre et Marie Curie, Paris, France UMW: Department of Respiratory Diseases, Aalborg University hospital; Respiratory Research Aalborg, Aalborg University, Aalborg, Denmark ZA, ME: Department of Respiratory Medicine, Allergology and Palliative Medicine, Institution for Clinical Sciences in Lund, Lund University, Lund, Sweden
Name and contact information for the trial sponsor {5b}	Region Skåne, 291 89 Kristianstad, Sweden
Role of sponsor {5c}	The funders have no influence on the trial design, conduct, or reporting. The coordinating PI and steering group are responsible for all decisions and all aspects of the trial

## Introduction

### Background and rationale {6a}

Long-term oxygen therapy is an established treatment that improves survival in people with chronic respiratory failure and severe hypoxemia due to chronic obstructive pulmonary disease (COPD) and interstitial lung disease (ILD) [1–4].

Oxygen therapy is usually administered through a nasal cannula using low flows (often 0.5–4 L/min), called low-flow oxygen therapy (LFOT), employing cold and dry gas [3, 4]. People with LFOT often experience side effects such as nose and upper/lower airways dryness, ulcerations, secretions, and phlegm stagnation, potentially contributing to a higher risk of exacerbations, hospitalisations, deterioration of health-related quality of life (HRQL), and premature death [5–8]. In addition, LFOT is the second most expensive health-care event (after hospitalisations) driving costs in people with respiratory disease such as COPD [9].

High-flow oxygen therapy (HFOT) is an emerging treatment alternative that provides humidified and heated gas via special equipment and a nasal cannula, specifically designed for high flows, at high flow rates (often 20–60 L/min). Beneficial physiological effects of HFOT include more effective oxygenation, improved wash-out of carbon dioxide, decreased dehydration, increased mucociliary clearance, improved respiratory mechanics, and higher treatment adherence [10, 11].

HFOT is believed to reduce the anatomical dead space and increase the wash out carbon dioxide [12]. Especially in COPD, dead space contributes to exercise intolerance and impaired ventilation, and subsequently HFOT may increase endurance and improve oxygen saturation during exercise [13].

The humidification and heating of the gas in HFOT is a very important component. Standard oxygen therapy affects the efficiency of the respiratory epithelium by decreasing humidity and temperature, which may lead to altered viscosity of respiratory secretions, bronchoconstriction, and reduced cilia movement and velocity of the mucociliary clearance [14, 15]. Impaired mucociliary clearance, in turn, is known to be associated with reduced lung compliance, increased airway resistance, and higher breathing work, and causes a risk of mucus plugging, alveolar atelectases, and infection [16, 17].

Finally, HFOT can also lead to improved pulmonary mechanics by reducing work of breathing, increasing lung compliance, improving gas exchange, and improving ventilation homogeneity [10].

Although HFOT is an open system with some leakage, it still provides a low positive airway pressure (PEP) of about 3 cm H2O, which may reduce inspiratory effort and work of breathing [11, 18]. In both healthy people and in patients with COPD, IPF, and IVA, HFOT has been shown to reduce respiratory rate, increase tidal volumes, decrease breathlessness, and improve ventilation homogeneity assessed using electrical impedance tomography, compared with low flow [11, 19].

As for the subjective experience, the most important positive effect of HFOT in acute hypoxic respiratory failure seems to be that HFOT compared with LFOT is associated with less dryness and less breathlessness [11, 20]. Reported side effects of prolonged HFOT after surgery and intubation are skin damage and erythema in the nasal philtrum, but this risk still seems lower than with NIV or CPAP [21, 22].

There are no obvious negative physiological effects of HFOT. However, it is important to emphasize that in spite of a small PEP, there is also still no evidence for replacing non-invasive ventilation as the gold standard treatment in acute hypercapnic respiratory failure [18, 19]. It is also of great interest that HFOT applied to tracheostomy makes no differences compared with LFOT, suggesting that the beneficial effect is mainly on the upper airways [23].

During the COVID-19 pandemic, HFOT was widely used with improved clinical outcomes among people with acute respiratory failure [24], and there is now an increasing interest in using HFOT for long-term treatment. In people with COPD and chronic hypoxic respiratory failure, a randomised controlled trial (RCT) from Denmark has reported a reduced risk of exacerbations, decreased breathlessness, and improved exercise tolerance and HRQL compared with regular LFOT [25]. In people with COPD and combined stable hypoxemia and hypercapnia, two Japanese studies reported a reduced exacerbation rate and improved HRQL [26, 27].

However, robust evidence from adequately powered multicentre studies evaluating critical outcomes such as mortality, hospitalisation rates, and health-economic impacts is notably lacking, underscoring the need for this trial. Furthermore, no trial has explored effects of HFOT in people with other diagnoses than COPD, such as ILD.

## Objectives {7}

The overall objective of this trial is to improve the evidence-based treatment in people with chronic respiratory failure due to COPD or ILD.

The primary objective is to test the effect of added HFOT as compared to usual LFOT on time to first hospitalisation or death during 1 year in people with oxygen-dependent chronic respiratory failure (CRF) due to COPD.

The secondary objectives are as follows:

- To evaluate treatment effects on other outcomes including respiratory symptoms, HRQL measured via validated questionnaires, and health-economics, and to explore the effects of HFOT in people with CRF due to ILD
- To assess adverse effects of HFOT compared with LFOT

## Trial design {8}

HILOT is a registry-based, randomized clinical trial (R-RCT) with the aim of investigating whether one of two treatment arms is superior to the other. There are two parallel groups, where the first treatment arm is existing standard treatment with LFOT at least 15 h/day. The second treatment arm also consists of oxygen at least 15 h/day, where LFOT is replaced by HFOT minimum during night-time. During daytime, the patient may choose between HFOT and LFOT.

**Fig. 1:**
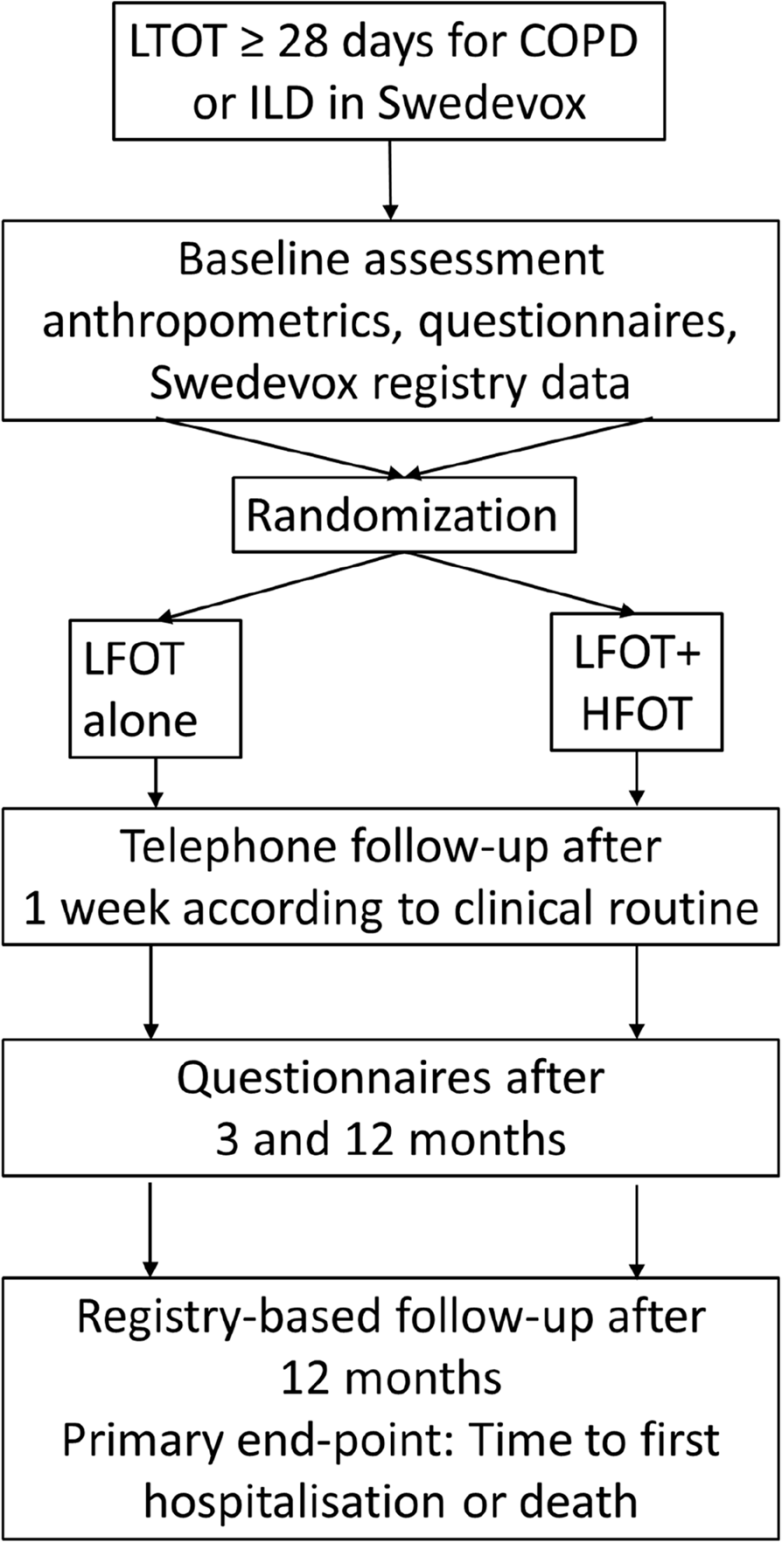
Trial outline. Abbreviations: COPD = Chronic Obstructive Pulmonary Disease, HFOT = High-Flow Oxygen Therapy, ILD = Interstitial Lung Disease, LFOT = Low-Flow Oxygen Therapy, LTOT = Long-term Oxygen Therapy

People with ongoing LFOT are randomised in a 1:1 ratio to standard treatment with LFOT or LFOT with added HFOT during nighttime and at the patient’s discretion daytime (Fig. 1). Randomisation will be performed between the treatment groups using a central, computerised, online procedure, stratified by main reason for the ongoing LFOT (COPD or ILD) and by study centre, in blocks of 2–8 participants. The randomised treatment group will not be blinded for the participants or the study staff for practical reasons but will be blinded for the statistician before the lock of the study database.

## Methods: participants, interventions, and outcomes

### Study setting {9}

Participants are recruited from ten secondary care centres in Sweden, connected to Swedevox: Umeå, Uppsala, Stockholm, Karlstad, Örebro, Linköping, Trollhättan, Gothenburg, Karlskrona, and Lund/Malmö. All sites and participants are connected to the oxygen arm of the Swedish National Registry of Respiratory Failure (Swedevox). Swedevox is a high-quality registry with 85–90% coverage, and with validated prospective national data collection on patients starting LTOT since 1987 [5, 28]. Swedevox data have previously successfully been used for registry-based controlled trials as well as cohort studies [29–32]. In the present study, the register is used for recruitment and baseline data. People registered in Swedevox due to ongoing therapeutic LFOT due to COPD or ILD since ≥ 28 days which is prescribed to be used ≥ 15 h per day are invited for screening. The primary study population is planned to be 270 people with CRF due to physician-diagnosed COPD. We also aim to include an exploratory cohort of 40 people with CRF due to ILD.

### Eligibility criteria {10}

Inclusion criteria are as follows:

- Age 40 years or older.
- Ongoing LFOT, prescribed for at least 15 h per day, and for a minimum of 28 days as registered in Swedevox.
- COPD or ILD as the main underlying reason for LFOT.
- Oxygen concentrator as a stationary oxygen source in the home including night-time.
- Body mass index (BMI) < 35 kg/m^2^ (to reduce potential confounding related to obesity-associated respiratory issues and sleep apnea).

Exclusion criteria are as follows:

- Current or previous treatment with home HFOT.
- Current treatment with home mechanical ventilation.
- Current treatment with home continuous positive airway pressure (CPAP).
- Hospitalised during the last 2 weeks.
- Current smoking or contact with flames.
- Self-reported average use of the LFOT < 15 h per day (24 h).
- PaCO2 (breathing air at rest) > 8 kPa.
- Strong clinical suspicion of obstructive sleep apnoea or obesity hypoventilation syndrome (as judged by the responsible staff).
- Inability to participate in the study procedures (as judged by the staff).
- Not eligible for continuing LFOT due to other reason (as judged by the staff).
- Expected survival less than 3 months (as judged by the staff).

### Who will take informed consent? {26a}

Participants will receive oral and written study information by the local primary investigator (PI), including that taking part is entirely voluntary and that they can withdraw from the study whenever they want without this affecting the usual care. Written informed consent will be obtained before study assessments and randomisation.

### Additional consent provisions for collection and use of participant data and biological specimens {26b}

Not applicable.

## Interventions

### Explanation for the choice of comparators {6b}

The intervention in this study is added nocturnal HFOT during sleep and at the patient’s discretion daytime, with standard LTOT treatment (LFOT) the rest of the time up to a minimum of 15 h oxygen per day. The comparison arm is continued usual care with LFOT at least 15 h/day, in accordance with clinical routine.

The investigational device used in this study is the system ResMed Lumis HFT and the AcuCare HFNC Cannula (Ref. Clinical Manual, © 2021 ResMed. 288280/4 2021–05). Lumis HFT delivers warmed and humidified respiratory gases to spontaneously breathing patients through a high flow nasal cannula, the AcuCare HFNC. The operating flow range of Lumis HFT is 15–40 L/min, and tube temperature and humidity can be adjusted. The Lumis HFT user guide, including technical specifications, is provided as supplemental material.

### Intervention description {11a}

At the start of Lumis-HFT, the device is connected to a power source. The humidifier is filled with water to the marker of maximum water level and placed at the side of the Lumis-HFT device, and the air hose is connected to the device. The ordinary LFOT concentrator is set in an off mode and is connected to the Lumis-HFT device. The Lumis HFT device is started and heated for 10–20 min. The device temperature is started at 34 °C, with an oxygen flow at 20 L/min and humidification at level 3 based on standard clinical guidelines to optimise patient comfort and adherence. After applying the cannula on the patient, the treatment is started by pushing the on-button at the Lumis HFT device, followed by starting the oxygen concentrator.

The oxygen flow default is 20 L/min, but if tolerated by the patient, the flow may be increased up to 30 or a maximum of 40 L/min. The oxygen fraction is titrated to reach the previously aimed saturation for standard LFOT, according to the Swedevox recommendations corresponding to PaO2 ≥ 8.0 kPa. The temperature aim is 37 °C, but may be lowered back to 34 or even 31 °C. If needed, the humidification may also be lowered to level 2 or increased to level 4 or 5.

### Criteria for discontinuing or modifying allocated interventions {11b}

Participants will be withdrawn if the trial treatment is judged to be harmful or carry unacceptable risk for harm, or at any time at the discretion of the participant. The trial treatment can be changed or discontinued at the discretion of the responsible physician or participant, which is not a reason for withdrawal from the trial.

Trial participation is completely voluntary and participants who wish to do so can withdraw at any time. Participants who want to withdraw will be asked which trial procedures they want to withdraw from, and whether they consent to be included in parts of the follow-up, including the registry-based endpoints.

### Strategies to improve adherence to interventions {11c}

At the telephone follow-up at one week (range 4 days) after randomisation, and in the questionnaires at 3 and 12 months, the patient is asked to report average daily oxygen and HFOT utilisation. Data on HFOT utilisation will also be collected from centres using the remote home-monitoring system AirView and from staff-reported questionnaires at 3 and 12 months (each to be filled in within ± 4 weeks). The information will be used to assess whether additional follow-up visits or calls are needed to improve adherence.

### Relevant concomitant care permitted or prohibited during the trial {11d}

This is a pragmatic academic trial investigating the addition of high-flow oxygen to standard treatment. All concomitant care is in accordance with clinical practice.

### Provisions for post-trial care {30}

After completion of the trial, the responsible clinician and the participant will decide if they would like to continue with HFOT treatment according to clinical routines. All participants are insured within the general Swedish Patient Insurance.

### Outcomes {12}

The primary outcome is time to first hospitalisation or death from all causes during 1 year after randomisation in people with COPD (at 1 year, assessed using nationwide registries).

Secondary outcomes are as follows:

- Time to first all-cause hospitalisation or all-cause death in people with ILD (at 1 year after randomisation, assessed using nationwide registries).
- Rate of hospitalisations or death from all causes (at 1 year).
- Hospitalisation rate from all causes (1-year and long-term).
- Hospitalisation rate from respiratory disease (1-year and long-term).
- Hospitalisation rate from cardiovascular disease (1-year and long-term).
- Number of hospitalised days (all-cause/respiratory disease/cardiovascular disease) (at 1 year and long-term).
- Number of admissions to intensive care unit (ICU) (at 1 year and long-term).
- Number of days in ICU care (at 1 year and long-term).
- The mortality rate from all causes (at 1 year and long-term).
- The mortality rate from respiratory disease (at 1 year and long term).
- The mortality rate from cardiovascular disease (at 1 year and long-term).
- Time to first exacerbation after randomisation, defined as time to first dispensed antibiotics and/or oral corticosteroids, emergency department visit, or hospitalisation for exacerbation (at 1 year).
- Number of exacerbations of all severity (at 1 year).
- Incidence of cardiovascular disease (at 1 year and long-term).
- Need for home mechanical ventilation (at 1 year and long-term).
- Rate of LFOT withdrawal (at 1 year and long-term).
- Safety and tolerability (adverse events at 3 and 12 months).
- Cost-effectiveness (at 1 year and long-term).
- HRQL (at 3 and 12 months).
- Symptoms (dyspnea, cough, phlegm, upper airway symptoms, fatigue, including nighttime symptoms, sleep) (at 3 and 12 months).
- Factors associated with the net clinical benefit of the HFOT, including the daily usage time.
- Patients’ perceptions of oxygen therapy and related factors.
- Adverse events as safety endpoints.

The secondary outcomes will be evaluated in the following populations: (1) people with COPD; (2) people with ILD; and (3) all people. All analyses will be performed primarily by randomised treatment group (intention to treat; ITT), and secondarily by treatment used (per protocol; PP).

### Participant timeline {13}

**Fig. 2:**
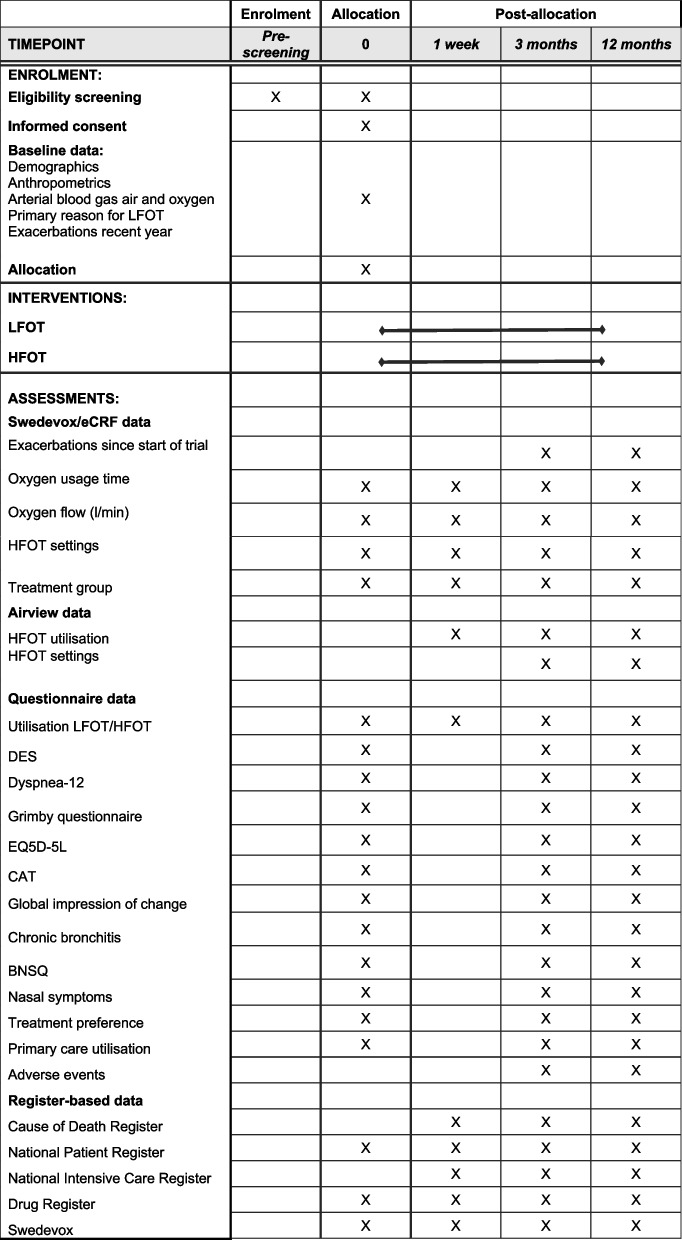
Schedule of enrolment, interventions, and assessments. Abbreviations: BNSQ = Basic Nordic Sleep Questionnaire, CAT = Chronic Obstructive Pulmonary Disease Assessment Test, DES = Dyspnea Exertion Scale, eCRF = electronic Case Report File, EQ 5D 5L = EuroQol 5 Dimension 5 Levels, HFOT = High-Flow Oxygen Therapy, LFOT = Low-Flow Oxygen Therapy

The time schedule of enrolment, interventions, follow-up, and assessments in the main trial is summarised in a SPIRIT figure (Fig. 2). In addition, long-term registry data withdrawal is planned.

### Sample size {14}

The sample size will be 270 randomised people with COPD and with data on the primary outcome. This sample size was determined to provide 80% power to detect a clinically meaningful between-group difference in the primary outcome (time to first hospitalisation or death from all causes during 1 year) of hazard ratio 0.67 in people with COPD, using a two-sided test, assuming equal allocation between the treatment groups, a 1-year rate of the primary outcome of 0.74 in the target population in the Swedevox registry; and a false positive rate (alpha) of 0.05 [33].

### Recruitment {15}

People with ongoing LFOT in the Swedevox registry are identified and screened for enrolment at the participating centres. In accordance with Swedish legislation and regulations, people starting LFOT are given written information about Swedevox including the choice to opt out from registration in Swedevox or for their data to be removed from the register at any time. People who do not opt out are registered in Swedevox, which has a high and stable nationwide coverage of about 85% of people starting LFOT in Sweden [5]. All people at participating units who are using LFOT since at least 28 days in the Swedevox registry will be screened for participation in the study.

## Assignment of interventions: allocation

### Sequence generation {16a}

Randomisation is performed within the web-system REDCap, a combined electronic Case Report File (eCRF) and randomisation module. The allocation is generated in blocks between 2 and 8, to reduce predictability of the sequence to the investigator.

### Concealment mechanism {16b}

The randomisation is computerised within the web-system REDCap, which means that the allocation is fully concealed until randomisation is performed.

### Implementation {16c}

The allocation sequence is generated by the web-system REDCap, and the local PI will enroll people and assign them to intervention according to randomization. 

## Assignment of interventions: blinding

### Who will be blinded {17a}

Due to the nature of the intervention (distinctive HFOT device setup), blinding of people and clinical staff is not feasible. However, the statistical analyst will remain blinded to allocation until database lock to minimise bias.

### Procedure for unblinding if needed {17b}

Non-applicable.

## Data collection and management

### Plans for assessment and collection of outcomes {18a}

Outcome data will be obtained from patient questionnaires at baseline, 3 and 12 months, from Swedevox, from trial specific data entered in the eCRF by the PIs, and from national registries. In centres using the remote home-monitoring system AirView, data on HFOT utilisation will be used.

The questionnaires will include questions on oxygen and HFOT usage, patient preference for continuing the treatment, utilisation of primary care and domestic service, adverse events, and the number of exacerbations since the start of the trial. Symptoms included in the questionnaires are breathlessness using a modified Dyspnoea Exertion Scale (DES) [34] and the Dyspnoea-12 questionnaire [35, 36], cough and phlegm assessed by relevant items in the COPD Assessment Test (CAT) [37], sleep complaints using a modified Basic Nordic Sleep Questionnaire (BNSQ) [38], nasal symptoms, and subjective perception of change in health status assessed with a modified Global Impression of Change (GIC) scale [39]. Physical activity is reported for the previous month using a modified Grimby activity questionnaire [40, 41], and HRQL is evaluated using the CAT [37] and EuroQol Five Dimensions Five Levels (EQ5D-5D-5L) [42].

Data from Swedevox will be used for characterising the people and their oxygen treatment, and for assessment of rate and reason of LFOT withdrawal during follow-up. The ventilator arm of the Swedevox registry will be used to assess the incidence of long-term home mechanical ventilator usage.

If applicable, data on HFOT utilisation will be collected from centres using Airview at 1 week, 3 and 12 months on: average usage time, daytime and nighttime use, days of use, and days of use ≥ 4 h/24 h. Data on oxygen flow and HFOT settings will be collected from staff-reported questionnaires at 3 and 12 months.

Data from the National Patient Registry from 1 January 1997 to end of trial for all in and outpatient care will be used to assess comorbidities at baseline, and time to, rate, and reason of hospitalisation and incident conditions and procedures during follow-up. Data from the Drug Registry on all dispensed outpatient prescriptions will be obtained from 1 year before the date of randomisation until the end of follow-up in the study. Data from the Intensive Care Registry on length of stay, procedures, treatments, complications, and death will be obtained to assess ICU care from 365 days before randomisation throughout follow-up. Data from the Cause of Death Register will be used to assess the date, place, whether autopsy was performed, and the causes of death throughout follow-up.

### Plans to promote participant retention and complete follow-up {18b}

Trial participation is completely voluntary, and people can withdraw at any time. People who want to withdraw will be asked which trial procedures they want to withdraw from, and whether they consent to be included in the follow-up of the registry-based endpoints. The trial treatment can be changed or discontinued at the discretion of the responsible physician or patient, which is not a reason for withdrawal from the trial. People who change or discontinue their treatment will be included in the analyses in accordance with randomised group (intention-to-treat) and per protocol (actual treatment group).

### Data management {19}

All data will be registered, managed, and stored in a manner that enables correct reporting, interpretation, and verification.

We will use an eCRF which allows for proper audit trial for all entries and changes to data. Descriptions of the methods for data entry and collection, including procedures for verification and validation, will be specified in the study-specific data management plan (DMP). All deviations from the DMP and other information of importance for the study outcome will be reported in the clean file form. Each site keeps a source data location list, identifying where source data are. Data collected from different registers will not be manually entered into the eCRF by site. The principal investigators or authorised designees shall, after verification of data entered by site, lock the data for further editing. By locking, the responsible investigator ensures that all data are correct and complete.

Procedures for data review, database cleaning, and issuing and resolving data queries will be specified in the study specific DMP. After data verification, validation, and all queries are resolved for all people, the database will be closed. All access will be withdrawn except for data export. All handling of all detected errors after database lock will be documented.

The PI and sponsor will maintain the essential clinical investigation documents in the investigation site files archive and sponsor files archive, respectively. The sponsor shall keep all documentation and data for at least 10 years after the trial has ended. The PI will archive all local investigation documentation for at least 10 years or as long as stipulated by the local institution.

### Confidentiality {27}

People who participate in the clinical investigation are coded with a specific clinical investigation identification number. All people are registered in a subject identification list (subject enrolment and identification list) that connects the subject’s name and personal number with a clinical investigation identification number. All data are entered into the eCRF, where access is allowed only with two-step authentication. Paper forms and questionnaires are saved in files that are stored in a locked area. The PI will archive all local investigation documentation for at least 10 years or as long as stipulated by the local institution.

The Swedish Social Security Number and date of randomisation in the trial will be sent securely to the trial unit Blekinge Tekniska Högskola (BTH; Blekinge Institute of Technology) that sends out and administers the patient questionnaires, which will be separate from the trial sponsor and monitor(s). The questionnaires will be posted to the patient at 3 and 12 months (± 2 weeks) after randomisation. All information collected by the sponsor will be pseudonymised and identified with Study ID, with a code list with study IDs and the corresponding Swedish Social Security Numbers safely stored separately.

### Plans for collection, laboratory evaluation, and storage of biological specimens for genetic or molecular analysis in this trial/future use {33}

Non-applicable.

## Statistical methods

### Statistical methods for primary and secondary outcomes {20a}

A detailed statistical analysis plan (SAP) will be established and published in collaboration with a biostatistician before the lock of the database for the primary analysis and before performing each secondary analysis.

The population for efficacy analyses is defined as all randomised people who did not withdraw their consent to be included in the outcome analyses (ITT population). The population for safety analyses is defined as all randomised people who started their randomised treatment (safety population). All analyses will be performed primarily by randomised treatment group (ITT) and secondarily by treatment used (PP).

Data will be summarised using mean with standard deviation and median with range and interquartile range for continuous variables with normal and skewed distributions, respectively. Categorical variables will be expressed as frequencies and percentages. Differences between groups will be analysed using Student’s *t*-tests (for normally distributed continuous outcomes), chi2 tests (for dichotomous outcomes), and Wilcoxon’s rank sum test (for ordinal and non-normally distributed data).

Outcomes will be compared between the treatment groups using multivariable regression models, expressed as point estimates with 95% confidence intervals. The primary outcome will be analysed using Cox proportional-hazards regression. Secondary outcomes will be analysed using appropriate statistical methods: logistic regression for categorical outcomes, Cox regression for standard survival analysis, Fine–Gray regression for competing risk scenarios, and linear regression for continuous outcomes, ensuring robust and context-appropriate analysis. Mixed regression models with random effects will be used to analyse outcomes measured in the same individual at several time points to account for repeated measurements. The outcome analyses will be adjusted for age and sex, the stratification variables, number of hospitalisations during the 12 months before baseline, and time from starting LFOT in Swedevox. Statistical significance will be defined as a two-sided *p*-value < 0.05.

### Interim analyses {21b}

No interim analyses will be performed, but the final number of randomised people needed to reach the target sample size will be checked before closure of trial enrolment, to ensure adequate power in the analysis of the primary outcome.

### Methods for additional analyses (e.g. subgroup analyses) {20b}

People with COPD and ILD will be analysed separately. If the projected sample size is not reached, all randomised people can be analysed as a secondary analysis, which will be detailed in the SAP.

### Methods in analysis to handle protocol non-adherence and any statistical methods to handle missing data {20c}

Missing values of predictors and confounders will be imputed by multiple imputation approaches.

Any trial deviations, including reportion deviations, will be specified in the SAP before each analysis.

### Plans to give access to the full protocol, participant level-data and statistical code {31c}

All upcoming manuscripts will be published with open access.

Data cannot be made freely available as they are subject to privacy in accordance with the Swedish Public Access to Information and Secrecy Act. Requests for data can be sent to the corresponding author and are available after approval from the Swedish Ethical Board (registrator@etikprovning.se) for researchers who meet the criteria for access to confidential data. Authors may be contacted to aid with the data access from the Ethical Board.

## Oversight and monitoring

### Composition of the coordinating centre and trial steering committee {5d}

The steering committee, including all co-authors of the protocol paper, is responsible for creating the protocol and standard operating procedures, and for follow-up meetings during the trial. The participants are all PIs and/or researchers with a special interest in the area. The research unit Clinical Studies Sweden Forum South is the coordinating centre and is responsible for agreements, start meetings support, and monitoring of the trial sites. A representative from ResMed is participating in all steering group meetings to support practical discussion about the high-flow device.

### Composition of the data monitoring committee, its role and reporting structure {21a}

The clinical investigation will be monitored by an independent monitor before the clinical investigation begins, during the clinical investigation conduct, and after the clinical investigation has been completed, to ensure that the clinical investigation is carried out according to the clinical investigation plan (CIP) and that data are collected, documented, and reported according to SS–EN ISO 14155:2020 and applicable ethical and regulatory requirements. Monitoring is performed as per the investigation’s risk analysis and monitoring plan and is intended to ensure that the subject’s rights, safety, and well-being are met as well as data in the CRF are complete, correct, and consistent with the source data.

### Adverse event reporting and harms {22}

As the LUMIS HFT device is an approved and CE-marked product, only unexpected events will be reported. According to the manufacturer, drying of the nose, mouth, or throat, nosebleed, bloating, and skin rashes may arise during treatment, but no serious adverse events have been recorded with HFOT in previous studies [13–15]. However, people should be asked to report unusual chest pain, severe headache, or increased breathlessness to their prescribing physician. An acute upper respiratory tract infection may require temporary discontinuation of treatment. Known side effects related to the trial treatments or underlying diseases will not be reported in the e-CRF.

Adverse events will be assessed at 1-week telephone follow-up, from patient register data on hospitalisations, from patient questionnaires at 3 and 12 months, and at the patient’s clinical visits according to clinical routines.

### Frequency and plans for auditing trial conduct {23}

Auditing of the trial takes place at the incentive of the Swedish Medical Product Agency (MPA). The MPA may suspend or prematurely terminate the clinical investigation at the applicable investigation sites.

### Plans for communicating important protocol amendments to relevant parties (e.g. trial participants, ethical committees) {25}

Amendments to the CIP will be agreed upon between the coordinating investigator and the Sponsor. Substantial modifications must be approved by the Swedish Ethical Review Authority and/or the Swedish Medical Products Agency (as applicable) before implementation. Investigator(s) are not allowed to deviate from the CIP except if it is for the protection of the subject’s rights, safety, or well-being under emergency circumstances. All such deviations shall be documented and reported to the Sponsor, the Swedish Medical Products Agency and/or the Swedish Ethical Review Authority (as applicable) as soon as possible. All deviations shall be documented with an explanation and reported to the Sponsor. Deviations will be reviewed by the Sponsor and reported to the appropriate regulatory bodies as required.

## Dissemination plans {31a}

The project will result in several publications in peer-reviewed international journals on: (1) trial design; (2) primary and main secondary outcomes; (3) health economic analysis; (4) additional secondary outcomes including in people with ILD; (5) correlational analyses of predictors of net clinical benefit; (6) systematic review and meta-analysis with all available data in this field; and (7) long-term registry follow-up. The results of the study are expected to be implemented in national and international clinical guidelines on home oxygen therapy.

## Discussion

This is the largest multi-centre RCT to assess the benefits of HFOT to date, and the first to include people with ILD. The study will have power to assess mortality and hospitalisation as a primary outcome and health-economics as a secondary outcome. Although LFOT is an established treatment that improves survival in people with chronic respiratory failure and severe hypoxemia [1, 2], the risk of hospitalisation and death in people with LFOT is very high [5, 8], and it is of great importance to assess interventions that may improve the prognosis. In addition, people with LFOT experience a high burden of local side effects with an impact on their HRQL [6], and HFOT could potentially decrease these problems.

The trial started with a pilot phase at the centres in Örebro and Karlskrona, to evaluate feasibility and therapy adherence. Early challenges were identified, particularly related to patient adherence due to device-related discomfort, as the majority of people randomised to HFOT were non-adherent and chose to end the treatment. The most common reasons were difficulties in sleeping due to discomfort and noise from the device. As a consequence, adjustments in initial flow settings and patient education strategies were made. The standard procedure was changed to aim for an initial lower oxygen flow of 20 L/min to get used to the high oxygen flow and to preferably use a somewhat larger nasal cannula to reduce the sound from the gas flow. In addition, a notification that it may take a couple of weeks to adjust to the new treatment was added to the standard information at the baseline visit.

Until end of spring 2025, start meetings have been held at the remaining sites, and the study is now recruiting at all ten sites.

## Trial status

The study is ongoing, present protocol version nr 1.1, dated 21 of February 2025. First recruitment was performed on 10th of June 2024. The study is expected to be finished earliest in 2028.

## Supplementary Information

Supplementary Material 1.

## Data Availability

The primary investigator and the project statistician will have full access to the final trial dataset. Data will not be shared with other investigators outside the sponsor Region Skåne unless preceded by a contractual agreement.

## Abbreviations

BMI: Body mass index
BNSQ: Basic Nordic Sleep Questionnaire
CAT: Chronic Obstructive Pulmonary Disease Assessment Test
CIP: Clinical investigation plan
COPD: Chronic obstructive pulmonary disease
CPAP: Continuous positive airway pressure
CRF: Chronic respiratory failure
DES: Dyspnoea Exertion Scale
DMP: Data management plan
eCRF: Electronic case report file
EQ-5D-5L: EuroQol-5 dimension- 5 levels
HFOT: High-flow oxygen therapy
HILOT: Effect of HIgh-flow Therapy in Long-term Oxygen Therapy
HRQL: Health-related quality of life
ILD: Interstitial lung disease
ITT: Intention to treat
LFOT: Low-flow oxygen therapy
MPA: Medical product agency
PI: Primary investigator
PP: Per protocol
OHS: Obesity hypoventilation syndrome
OSA: Obstructive sleep apnoea
RCT: Randomised controlled trial
R-RCT: Registry-based randomised clinical trial
SAP: Statistical analysis plan
